# A rapid response of lung squamous cell carcinoma following treatment with sintilimab combined with recombinant humane endostatin injection and nab-paclitaxel in an elderly patient: A case report

**DOI:** 10.1097/MD.0000000000026801

**Published:** 2021-08-06

**Authors:** Yueyu Fang, Hui Sun, Yi Chen, Nanyuan Jiang, Lianhua Ji, Junfeng Shi

**Affiliations:** a Department of Oncology, Nanjing Pukou Central Hospital, Pukou Branch Hospital of Jiangsu Province Hospital, Nanjing, People's Republic of China; b Department of Oncology, Nanjing First Hospital, Nanjing Medical University, Nanjing, People's Republic of China.

**Keywords:** endostar, lung squamous cell carcinoma, programmed death 1 inhibitor, recombinant humane endostatin injection, sintilimab

## Abstract

**Rationale:**

At present, the prognosis of patients with giant lung squamous cell carcinoma (LSCC) is poor, and there is no safe and effective treatment for elderly patients with large LSCC.

**Patient concerns:**

Here, we reported a 77-year-old man admitted to the hospital with cough for 3 months and significant chest pain. Computed tomography (CT) imaging showed a large mass in the left lung with pleural effusion.

**Diagnoses:**

Chest CT scan revealed a 12.5 cm × 7.3 cm mass in the left upper lobe adjacent to the pulmonary vein, with left pleural effusion. Pulmonary tumor markers were significantly elevated, and CT-guided percutaneous lung mass biopsy specimens showed LSCC.

**Interventions:**

After diagnosis, the patient was treated with sintilimab combined with endostar and nab-paclitaxel. After 2 cycles of treatment, the lung mass in the patient shrank rapidly and the clinical symptoms were relieved.

**Outcomes:**

The patient's tumor dramatically shrank, and the pleural effusion was decreased after 4 cycles of treatment without any adverse effects. Meanwhile, the high-level tumor marker resumed normal.

**Lessons:**

Sintilimab combined with endostar and nab-paclitaxel may be a good treatment option for lung squamous cell cancer, especially for that in elderly patients.

## 1 Introduction

Lung cancer is one of the most common malignancies and the leading cause of cancer death worldwide.^[1]^ It can be divided into small cell lung cancer (SCLC) and non-small cell lung cancer (NSCLC). NSCLC accounts for about 80–85% of lung cancer, and lung squamous cell carcinoma (LSCC) accounts for about 25–30% of NSCLC^[2]^ After a combination of surgery, radiation and chemotherapy, the 5-year survival rate of patients with advanced LSCC is only 5%.^[3]^ Programmed death 1 (PD-1) is an inhibitory receptor expressed on T cells, and its ligands include programmed death ligand 1 (PD-L1) and programmed death ligand 2 (PD-L2). PD-1/PD-L1 binding activates the immune checkpoint pathway and inhibits T-cell-mediated immune responses.^[4]^ Sintilimab, a PD-1 inhibitor developed by Innovent Biologics and Eli Lilly and Company, has been used in combination with pemetrexed and platinum as the first-line therapy for patients with advanced or recurrent non-squamous NSCLC under the approval of the National Medical Products Administration of China.^[5]^

Endostatin is a new targeted therapeutic agent, which can inhibit the proliferation of vascular endothelium, induce the apoptosis of endothelial cells, block the signaling pathway of vascular endothelial growth factor (VEGF) and down-regulate the expression of genes related to angiogenesis to play an anti-tumor role.^[6]^ In 2005, the State Food and Drug Administration of China approved the application of the modified recombinant human endostatin endostar for the treatment of NSCLC. In several studies, endostar combined with chemotherapy has shown good objective response and high safety in the treatment of patients with advanced LSCC.^[7–10]^

Here, we report on an elderly Chinese patient with stage IV LSCC who responded significantly to 4 cycles of chemotherapy combined with sintilimab and endostar therapy.

## 2 Case report

**Figure 1:**
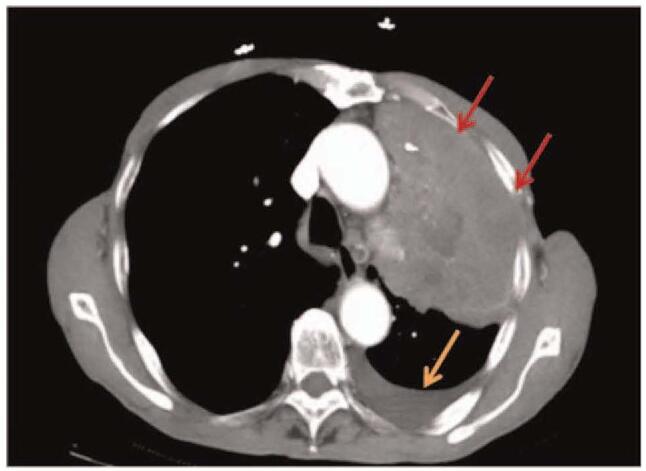
Computed tomography at the first presentation showing a 12.5-cm massive pulmonary tumor in the left upper lobe (Mediastinal window). The red arrows indicated lung masses and the yellow arrows indicated pleural effusion.

**Figure 2:**
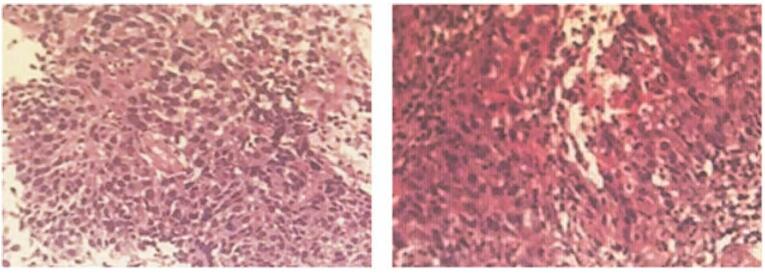
Histological examination of lung biopsy specimens obtained from the left pulmonary mass revealing squamous cell carcinoma.

**Figure 3:**
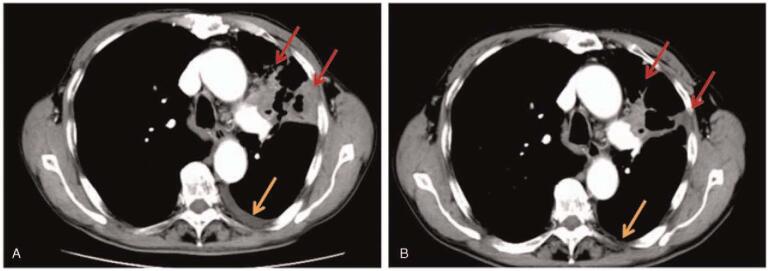
Chest CT images obtained after 2 course (A) and 4 course (B) of treatment with sintilimab combined with endostar and nab-paclitaxel. The red arrows indicated lung masses and the yellow arrows indicated pleural effusion. CT = computed tomography.

A 77-year-old man was admitted to our hospital with a 3-month history of cough and obvious chest pain. He smoked 2 packs a day for fifty years, with a 30-year history of chronic bronchitis. His Eastern Cooperative Oncology Group performance status was 1. Chest computed tomography scan on October 30, 2020 showed a lung mass of 12.5 cm × 7.3 cm in the left upper lobe adjacent to the pulmonary vein, accompanied by left pleural effusion (Fig. 1), and the mass did not metastasize to the abdomen, brain, or bone. Pulmonary tumor marker test on November 12, 2020 revealed carcino-embryonic antigen = 30.22 ng/mL, squamous cell carcinoma antigen = 11.1 ng/mL, neuron specific enolase = 46 ng/mL and cytokeratins > 500 ng/mL. All other laboratory data obtained from blood routine examination and liver and renal function tests were within the normal range. Histological examination of CT-guided percutaneous lung biopsy specimens from the left lung mass confirmed LSCC (Fig. 2). The patient refused genetic testing, so the expression of PD-L1 was unknown. According to the 8th edition lung cancer stage classification, his disease was clinically staged as IVa (T3N2M1a) and was therefore inoperable. Subsequently, the combined therapy using 30 mg endostar type IV collagen for 24 hours on days 1–7, 200 mg sintilimab on day 3, and 300 mg nab-paclitaxel on day 3, a first-line treatment, was adopted. After 2 cycles of treatment, the disease was evaluated on January 5, 2021 and characterized as a partial response (PR) based on the Response Evaluation Criteria In Solid Tumors 1.1 (Fig. 3A). Due to the effective response, the patient received 2 more cycles of treatment. After the 4th cycle of treatment, the tumor shrank significantly and the pleural effusion was decreased, as evidenced on March 2, 2021 (Fig. 3B). Moreover, the tumor marker test revealed carcino-embryonic antigen = 6.53 ng/mL, squamous cell carcinoma antigen = 0.58 ng/mL, neuron specific enolase = 11.9 ng/mL, and cytokeratins = 2.28 ng/mL. Up to now, the disease remains stable. During the treatment, cough and chest pain of the patient were significantly eased, with no significant adverse effects (AEs).

## 3 Discussion

Lung cancer is the most common malignancy with high morbidity and mortality.^[1]^ LSCC is a common pathological type of NSCLC. Due to hidden symptoms of LSCC in the early stage, most LSCC patients have been already in the advanced stage when diagnosed.^[11]^ Although important advances have been made in molecular targeted therapies for NSCLC,^[12–14]^ the complexity of genetic alterations in LSCC has limited its application.^[15,16]^ Platinum-based doublet chemotherapy is the classic first-line treatment for LSCC.^[17,18]^ However, due to the limited efficacy and increasing toxicity, especially for elderly patients, we need to discover novel therapeutic approaches.

Recently, the emergence of immune checkpoint inhibitors has fundamentally changed the treatment landscape of NSCLC. Based on the KEYNOTE-407, the combination of pembrolizumab with carboplatin and nab-paclitaxel (or paclitaxel) to treat LSCC in first-line was approved by the Food and Drug Administration in 2019.^[19]^ However, the high cost of pembrolizumab limits its use in China. Sintilimab is a fully human IgG4 monoclonal antibody against PD-1 that blocks the interaction between PD-1 and its ligands and helps T cells restore their anti-tumor effects.^[20,21]^ Compared with pembrolizumab, sintilimab has similar antitumor effects, better safety, and more economical.^[22,23]^ In a phase Ib study, neoadjuvant sintilimab monotherapy in NSCLC resulted in an objective response rate (ORR) of 20% (8/40) and a disease control rate (DCR) of 90% (36/40), similar to those after pembrolizumab and nivolumab therapy.^[24,25]^ Another multicenter study involving 20 advanced LSCC patients in China showed satisfying efficacy and a high tolerance rate after the treatment of sintilimab combined with chemotherapy.^[26]^ Most recently, a randomized, double-blind, phase III study (ORIENT-11) involving 397 IIIB-IV non-squamous NSCLC patients with no previous systemic treatment has obtained a positive result. It was found that sintilimab combined with pemetrexed plus platinum significantly extended progression-free survival (PFS) compared with chemotherapy alone. Although the median overall survival (OS) was not achieved, the median PFS, ORR, and DCR in combined therapy group and chemotherapy group were 8.9 months versus 5.0 months, 51.9% (138/266) versus 29.8% (39/131), and 86.8% (231/266) versus 75.6% (99/131), respectively. No serious adverse reactions were found.^[5]^ Based on this study, sintilimab has been approved to be used for first-line therapy in patients with advanced non-squamous NSCLC. Although the application of sintilimab is still in the clinical trial phase, and its efficacy and safety in LSCC needs more tests, its application in treating LSCC will likely be approved in the near future.^[26,27]^ In one report, a 64-year-old woman diagnosed with initially unresectable squamous NSCLC achieved pathologic complete response and remained disease-free in the subsequent several-month follow-up after 3 cycles of the combined therapy of sintilimab plus nedaplatin and paclitaxel before surgery.^[28]^

Folkman first proposed that antiangiogenesis agents can inhibit tumor growth.^[29]^ Endostatin can significantly inhibit the proliferation and migration of vascular endothelial cells, leading to the apoptosis of vascular endothelial cells.^[6]^ As such, it can prevent tumor cells from receiving the nutrients necessary for growth and metastasis.^[6]^ Recombinant human endostatin (Endostar) is an antiangiogenesis agent that was developed independently in China. Preclinical and clinical studies have demonstrated its effectiveness in treating various types of cancers.^[30–32]^ A phase III, randomized, double-blind, placebo-controlled study compared the efficacy and safety of endostar plus vinorelbine-cisplatin (NP regimen) with placebo plus NP in advanced NSCLC patient. The results manifested that Endostar plus NP improved ORR (35.4% vs 19.5%, *P* = .0003) and DCR (73.3% vs 64.0%, *P* = .035). Furthermore, endostar plus NP prolonged time to progression (TTP; 6.3 months vs 3.6 months, *P* < .001), OS (13.8 months vs 9.8 months, *P* < .0001) and increased quality of life score (QoL score; [54.4 ± 3.7] vs [53.4 ± 5.9], *P* = .0155).^[33]^ Multiple MATA (a method of individual studies for the purpose of integrating the findings) analyses also confirmed the effectiveness of endostar in combination with chemotherapy for advanced NSCLC.^[34,35]^

Antiangiogenesis agents can not only reverse the immunosuppressive effect caused by VEGF, but also normalize the tumor vascular system and promote the delivery of T cells and other immunoeffector molecules. In the meantime, immune checkpoint inhibitors can normalize the tumor vascular system by activating effector T cells, and increase the infiltration and killing function of effector T cells. Thus, immunotherapy combined with antiangiogenesis agents can form a positive feedback and mutually synergistic effect.^[36]^ Multiple phase I/II studies have shown that immunotherapy combined with antiangiogenesis agents displays anti-NSCLC activity. A multicohort, non-randomized, open-label, phase 1a/b trial assessed the safety and preliminary antitumor activity of ramucirumab (an immunoglobulin 1 VEGFR-2 antagonist) combined with pembrolizumab in patients with previously treated advanced NSCLC. The results showed that 85.2% of patients experienced at least one reduction of the lesion, with an ORR of 29.6%; PFS at 6 and 12 months were 66.7% and 44.4%, respectively; the OS at 6 and 12 months were 85.2% and 66.7%, respectively^[37]^ Another phase 1b study using sintilimab plus anlotinib (a multitarget antiangiogenic tyrosine kinase inhibitor) as first-line therapy in patients with advanced NSCLC showed ORR of 72.7% (95% CI: 49.8–89.3%) and DCR of 100% (95% CI: 84.6–100%). Median PFS was 15 months (95% CI: 8.3 m, not reached), and the 12-month PFS rate was 71.4% (95% CI: 47.2–86.0%).^[38]^

A large number of studies have revealed that PD-L1, tumor mutation burden and microsatellite high instability (MSI-H)/mismatch repair deficient (dMMR) can be used to predict the efficacy of immunotherapy, but they’re not perfect biomarkers.^[39]^ Although there are many studies exploring VEGF-A, VEGFR-2, fibroblast growth factor 2 (FGF-2), interleukin-6 (IL-6), IL-8, toxicity (hypertension and hand-foot syndrome), or specific angiogenesis-related genes in predicting the efficacy of antiangiogenesis agents, the results were almost always negative.^[40]^ To date, no biomarker has been found that can accurately predict the efficacy of immune combined antiangiogenesis.

The common AEs of sintilimab included pneumonia, diarrhea, colitis, hepatitis, nephritis, endocrinology diseases, skin AEs, infusion reactions, and other immune-related AEs.^[23]^ The most common clinical AEs of endostar are cardiac reactions. Rarer reactions mainly include skin and accessory allergic reactions and digestive tract reactions. In our case, no adverse events were observed, probably because of short treatment course and follow-up period.

## 4 Conclusion

In this study, an elderly patient was treated with sintilimab in combination with endostar and chemotherapy. To our surprise, after only 2 cycles of treatment, most of the huge lumps in the lungs shrank. Meanwhile, the AEs were tolerable. Our case report is the first report using sintilimab and endostar with nab-paclitaxel in advanced LSCC, with rapid response.

## Author contributions

**Data curation:** Lianhua Ji.

**Funding acquisition:** Junfeng Shi, Yi Chen.

**Methodology:** Junfeng Shi.

**Resources:** Yueyu Fang, Yi Chen, Nanyuan Jiang.

**Supervision:** Nanyuan Jiang.

**Writing – original draft:** Yueyu Fang.

**Writing – review & editing:** Junfeng Shi, Hui Sun.
